# Supplementary material for: Effects of anti-malarial prophylaxes on maternal transfer of Immunoglobulin-G (IgG) and association to immunity against Plasmodium falciparum infections among children in a Ugandan birth cohort
Source: PLoS One. 2023 Feb 22;18(2):e0277789. doi: 10.1371/journal.pone.0277789 (PMC9946240; doi:10.1371/journal.pone.0277789)
Supplement: S1 Appendix — (PDF) [file pone.0277789.s001.pdf]

MAKERERE UNIVERSITY
P.O. Box 7072 Kampala, Uganda
E-mail: biomedicalresearch62@gmail.com
Phone: 256752575050
Fax: 256 414 532204

WE BUILD FOR THE FUTURE

COLLEGE OF HEALTH SCIENCES
SCHOOL OF BIOMEDICAL SCIENCES
HIGHER DEGREES RESEARCH AND ETHICS COMMITTEE

23rd Feb 2018

File: SBS-342

To: Prof. Moses Kamya
Principal Investigator
School of Medicine
Makerere University College of Health Sciences

Category of review
[ ] Initial review
[X] Continuing review
[ ] Amendment
[ ] Termination of study
[ ] SAEs

In the matter concerning continuing review of a research proposal entitled **"Prevention of malaria in HIV- uninfected pregnant women and infants"**

The School of Biomedical Sciences Higher Degrees Research and Ethics Committee (**SBS-HDREC**) reviewed the progress report of the above study and found the report satisfactory and granted continuing **APPROVAL** valid until **1st March 2019.**

Please note that the annual report and the request for renewal where applicable should be submitted 6 weeks before expiry date of current approval.

Any problems of a serious nature related to the execution of the research protocol should be promptly reported to the **SBS-HDREC**, and any changes to the research protocol should not be implemented without approval from **SBS-HDREC**, except when necessary to eliminate apparent immediate hazards to the research participant(s).

Signed..................................

Dr. Erisa Mwaka
Chairperson, School of Biomedical Sciences Higher Degrees Research and Ethics Committee

MAKERERE UNIVERSITY
SCHOOL OF BIOMEDICAL SCIENCES
APPROVED
VALID UNTIL
01 MAR 2019
RESEARCH & ETHICS COMMETTEE
P. O. BOX 7072, KAMPALA
