# Supplementary material for: Effects of anti-malarial prophylaxes on maternal transfer of Immunoglobulin-G (IgG) and association to immunity against Plasmodium falciparum infections among children in a Ugandan birth cohort
Source: PLoS One. 2023 Feb 22;18(2):e0277789. doi: 10.1371/journal.pone.0277789 (PMC9946240; doi:10.1371/journal.pone.0277789)
Supplement: S2 Appendix — (PDF) [file pone.0277789.s002.pdf]

MAKERERE UNIVERSITY
P.O. Box 7072 Kampala, Uganda
E-mail: biomedicalresearch62@gmail.com
Tel:+ 256 757575050
Fax: 256 414 532204

COLLEGE OF HEALTH SCIENCES
SCHOOL OF BIOMEDICAL SCIENCES
HIGHER DEGREES RESEARCH AND ETHICS COMMITTEE

3rd April 2019

File: SBS-658

To: Mr. Okek Erick
Principal Investigator
Department of Immunology and Molecular Biology
Makerere University College of Health Sciences

Category of review
[X] Initial review
[ ] Continuing review
[ ] Amendment
[ ] Termination of study
[ ] SAEs

Decision of the School of Biomedical Sciences Higher Degrees Research and Ethics Committee (SBS-HDREC) following its 90th REC meeting held on 21st Feb 2019.

In the matter concerning the review of a research proposal entitled **"Maternal transfer of Immunogloublin-G (IgG) subtypes associated with Plasmodium Falciparum infections among infants in a Ugandan birth cohort"**

The investigator has met all the requirements as stated by **SBS-HDREC** and therefore, the protocol is **APPROVED.**

The approval granted includes all materials submitted by the investigator for SBS-HDREC review including;

1. Protocol version 3.0 April 2019

and is valid until **20th Feb 2020**

Any problems of a serious nature related to the execution of the research protocol should be promptly reported to the **SBS-HDREC**, and any changes to the research protocol should not be implemented without approval from **SBS-HDREC** except when necessary to eliminate apparent immediate hazards to the research participant(s)

Please note that the annual report and the request for renewal where applicable should be submitted to the **SBS-HDREC** office at least six (6) weeks before expiry date of approval.

You are required to register the research protocol with the Uganda National Council for Science and Technology (UNCST) for final clearance to undertake the study in Uganda.

Signed.....................................

Dr. Erisa Mwaka
Chair person, School of Biomedical Sciences Higher Degrees Research and Ethics Committee

MAKERERE UNIVERSITY
SCHOOL OF BIOMEDICAL SCIENCES
VALID UNTIL
20 FEB 2020
P.O. BOX 7072, KAMPALA
