# Supplementary material for: Effects of anti-malarial prophylaxes on maternal transfer of Immunoglobulin-G (IgG) and association to immunity against Plasmodium falciparum infections among children in a Ugandan birth cohort
Source: PLoS One. 2023 Feb 22;18(2):e0277789. doi: 10.1371/journal.pone.0277789 (PMC9946240; doi:10.1371/journal.pone.0277789)
Supplement: Attachment — Submitted filename: Finale;PLOS ONE rebuttal_tracked changes_moses_comments (1).doc [file pone.0277789.s008.doc]

**Response to gaps identified and guidance offered following submission of Manuscript to PLOS ONE publication.**

The authors of the manuscript would wish to sincerely appreciate your honest and candid feedback following the article submitted to PLOS ONE journal for reviews, edits and eventual publications. We have sat down as a team and made adjustments and formatted the manuscript to conform to your guidance and editorial reviews; details of gaps identified, and corresponding responses are detailed below

Comment #1, Editor

1.Please ensure that your manuscript meets PLOS ONE's style requirements, including those for file naming. The PLOS ONE style templates can be found at 
<https://journals.plos.org/plosone/s/file?id=wjVg/PLOSOne_formatting_sample_main_body.pdf> and 
<https://journals.plos.org/plosone/s/file?id=ba62/PLOSOne_formatting_sample_title_authors_affiliations.pdf>

**Response:** We take note of your guidance; the link shared has been so helpful in adjusting the manuscript to conform to PLOS ONE publication requirement. The revised manuscript re-submitted now meets standards for publication in PLOS ONE Journal. The revised Manuscript has been adjusted to conform to PLOS ONE publication

**Comment#2, Editor**:We note that the grant information you provided in the ‘Funding Information’ and ‘Financial Disclosure’ sections do not match

**Response:** . Thanks for the comment; information on the funding and financial disclosure section has been revised in the re-submitted manuscript

**Comment #3, Editor:** We note that you have stated that you will provide repository information for your data at acceptance. Should your manuscript be accepted for publication, we will hold it until you provide the relevant accession numbers or DOIs necessary to access your data. If you wish to make changes to your Data Availability statement, please describe these changes in your cover letter and we will update your Data Availability statement to reflect the information you provide.

**Response:** Thanks for the observation, the data availability statement has been revised to indicate that, all the data for the study will be available on request from the author upon publication of the manuscript. This information has been included in the revised cover letter

**Comment #4**, **Editor**: PLOS requires an ORCID iD for the corresponding author in Editorial Manager on papers submitted after December 6th, 2016. Please ensure that you have an ORCID iD and that it is validated in Editorial Manager. To do this, go to ‘Update my Information’ (in the upper left-hand corner of the main menu), and click on the Fetch/Validate link next to the ORCID field. This will take you to the ORCID site and allow you to create a new iD or authenticate a pre-existing iD in Editorial Manager. Please see the following video for instructions on linking an ORCID iD to your Editorial Manager account: <https://www.youtube.com/watch?v=_xcclfuvtxQ>

**Response:** Thanks for the guidance, the ORCID ID for the corresponding author (*: 0000-0002-2836-714* has been provided in the revised manuscript.

**Comment #5,** **Editor**: Please include your full ethics statement in the ‘Methods’ section of your manuscript file. In your statement, please include the full name of the IRB or ethics committee who approved or waived your study, as well as whether you obtained informed written or verbal consent. If consent was waived for your study, please include this information in your statement as well.

**Response**:. Our current study used samples that were collected from a previous study that was reviewed and cleared by School of Biomedical Sciences Research and Ethics Committee (SBS 114). The samples from primary study were collected after obtaining a written informed consent from study participants. This current study obtained ethical clearance from School of Biomedical sciences Research and Ethics Committee, College of Health Sciences Makerere University (SBS 1012). The archived samples used for this study, were de-identified prior to being accessed by the study team.

**Comment #6, Editor**: We note that Figure 1 in your submission contain [map/satellite] images which may be copyrighted. All PLOS content is published under the Creative Commons Attribution License (CC BY 4.0), which means that the manuscript, images, and Supporting Information files will be freely available online, and any third party is permitted to access, download, copy, distribute, and use these materials in any way, even commercially, with proper attribution. For these reasons, we cannot publish previously copyrighted maps or satellite images created using proprietary data, such as Google software (Google Maps, Street View, and Earth). For more information, see our copyright guidelines: <http://journals.plos.org/plosone/s/licenses-and-copyright>

**Response:** Thanks for the observation, however, fig1 was developed from the analysis of the data generated from our study. The figure 1 is not a map but was generated from the analysis of our study data.

**Comment #7, Editor**: Please include captions for your Supporting Information files at the end of your manuscript, and update any in-text citations to match accordingly. Please see our Supporting Information guidelines for more information: <http://journals.plos.org/plosone/s/supporting-information>.

**Response:** . Thank you for the observations; proposed corrections have been effected in the revised manuscript

**Comment #8 Editor**: Please amend the manuscript submission data (via Edit Submission) to include author Kiyimba Anthony, Arinaitwe Emmanuel, Nankabirwa Joaniter, and Kamya.R.Moses.

**Response:** Thanks for observation, the authors were omitted from the initial submission, but have been included in the revised manuscript.

**Comment#9 Editor**: Please ensure that you refer to Figure xxxxx in your text as, if accepted, production will need this reference to link the reader to the figure.

**Response**: This guidance is not very clear, there is no such figure as xxxxx in my text; However, we have referenced all the figures in the main text of the revised manuscript.

**Comment #10, Editor:** Please include captions for your Supporting Information files at the end of your manuscript, and update any in-text citations to match accordingly. Please see our Supporting Information guidelines for more information: <http://journals.plos.org/plosone/s/supporting-information>.

**Response**: Thanks for the comments; this has been effected in the revised manuscript.

Reviewer’s comments

Reviewer One:

**Comment#1: Reviewer 1:** The authors mentioned that “Increasing antibody titer against P.falciparum may be more of a
marker of exposure to previous malaria infections”. What does “previous malaria” mean here? Mothers got malaria during pregnancy. Doesn’t placenta malaria capture that information?

**Response**: Thank you for the comment, we acknowledged the lack of clarity in usage of the term “Previous malaria infections”; however, we intended to mean that the antibody titre levels observed amongst study participants were due to an exposure to *Plasmodium falciparum* parasites rather than being an indicator of protective immune response. The term “previous malaria” has been replaced by exposure to *Plasmodium falciparum* parasites.

**Comment#2; Reviewer 1**: For the survival analysis what if the placenta malaria status was used as the grouping variable instead of IgG levels?

**Response**: In our study, we sought to assess how malaria infections in pregnancy affects malaria episodes in children born to these mothers. Therefore, measurements of IgG level was done as a marker of malaria infection in children. The IgG levels is thus the most appropriate grouping variable. If we use placental malaria as a grouping variable, it would be difficult to test our hypothesis that malaria specific IgG levels protects against both infection and severe disease in the first few months of life.

**Comment#3; Reviewer 1**: Does “ANOVA in Box plot” means the data are plotted in box plot and comparisons were done using ANOVA? ANOVA compares means instead of medians. Because there are only two groups in the comparison, ANOVA is the same as t-test. Also, many of the log-IgG level seem to have a skewed distribution, a non-parametric test may be needed. Due to the relatively large number of comparisons (4 IgG subtypes and 15 antigens) P-values need to be adjusted for multiple testing.

**Response**:

Thanks for the comment, we have now analyzed the data using, Man-Whitney u test (a non-parametric test) has been used to compare means of IgG sub-type levels across DP and SP in the revised manuscript. Independent variable was treatment arm while dependent variable was IgG sub-type levels against different *P. falciparum* antigens.

**Comment #4; Reviewer 1:** In the result section about survival curves, hazard ratio (HR) was given at the end of the paragraph, but which IgG was this? Does the star in Figure 3 means p<0.05 between the two groups? If so, stars were seen in most of the subplots but only a few HRs were mentioned in the text. In addition, it will be interesting to fit a Cox regression model to assess the association while adjusting other covariates in the model. The low concentration group had a lower risk comparing to the higher concentration group. Shouldn’t the HR be <1 rather than >1. Please double check the text/numbers.

**Response:** Noted; it is true that hazard ratio stated is only for IgG1 because it is the IgG sub class that plays the most vital role in malaria immunology; reciprocal Hazard ratio is what has been presented explaining why values are more than one.

**Comment #5; Reviewer 1:** Are data on the mother’s social economic status (SES) available? If so, it would be interesting to include the SES variables in the regression models that predicting IgG levels and the Cox model to see if they play a role.

**Response:** Yes, Social Economic status has been captured; these includes wealth index category (poor, very poor, average, well off), Education status type of house construction, and use of long-lasting insecticide treated mosquito net. Previous studies that attempted to look at effects of social economic status of mothers on cord expression of IgG levels in newborn did not have significant findings or association between SES and cord IgG sub-class expression

**Reviewer 2:**

**Comment #1; Reviewer 2:** Authors can revise the title and mention the effect of treatment with SP and DP, instead of talking only about P. falciparum infection

**Response**: Thanks for the comment, our revised tittle now reads; *“Effects of anti-malarial prophylaxes on maternal transfer of Immunoglobulin-G (IgG) and association to Immunity against Plasmodium falciparum infections among Children in a Ugandan birth Cohort”*

**Comment #2 Abstract; Reviewer 2:** Choose between antibody and anti-body

**Response**: Authors have chosen to use “antibody” not “anti-body”; this has been corrected all throughout the revised manuscript

**Comment #3 Abstract, Reviewer 2**: Results: Specify the difference (high or low) in IgG4 between children from mothers on SP vs DP, same thing for EBA

**Comment #4 Abstract**,Reviewer 2: Conclusion: " increasing antibody titer against P.falciparum " in whom? Please specify

**Response:** Thanks for the comment. This has been corrected in the revised manuscript, ‘increasing antibody titer against *P. falciparum* in children’

**Comment #5, Introduction; Reviewer 2:**
- Antibody dependent cellular cytotoxicity (ADCC) - not antigen dependent cellular cytotoxicity
- Put space before the references or not. Please harmonize
- High expression of IgG4 has been associated with repeated exposure to specific…of IgG4: Please put reference

**Response**: Statement has been changed from **antigen** dependent cellular cytotoxicity to **antibody** dependent cellular cytotoxicity as guided by the reviewer.

**Comment #6: Reviewer 2:** Materials and methods
- Participants and data collection: Give enough details
- Describe preparation of buffer A, then B
- MagPix multiplex bead array assay: If available, please provide the IgG positivity threshold and specify how this threshold was determined
- The sample collection times for carrying out the survival tests were not detailed.

**Response:** Demographic and clinical details of study participants is captured in a previous study which was published <https://doi.org/10.1016/S0140-6736(18)32224-4>; it has been cited as well .Description of Preparation of buffer A was made first, that of buffer B then followed as guided by the reviewer. MagPix multiplexbead array assay: The plates were read on a Magpix platform (Luminex, USA), acquiring at least 50 beads/region/well. The results were expressed as median fluorescence intensity (MFI). The blank well MFI (background effects) was deducted from each well to determine the net MFI (IgG positive result). Sample collection for Kaplan Meir survival curve analysis

Primary study where samples for this study were retrieved from was collected during delivery (cord blood). Collection was done by the midwife, medical officer or obstetrician who conducted delivery or cesarian section. Average of 4mls of accompanying mother’s venous blood was collected as well in an EDTA tube (mother baby pair). Both cord and maternal blood were centrifuged to obtain serum, archived at appropriate temperature, and retrieved for this study. Archival was done in mother-baby pair arrangement, a random-access number generated by the computer was used to track samples in the biobank. Venous blood samples were collected from infants at different time points during first year of life as and when malaria related signs and symptoms such as fever, vomiting or chills appear. Blood samples obtained was prepared and examined for presence of *P.falciparum* parasites; balance archived and used for future study like this one. Obtaining samples at different time points allowed for estimation of antibody decay rates.

# **Preparation of buffer A**

Buffer A was prepared as discussed previously, but briefly, 1L of phosphate buffer solution was measured and put in a conical flask after which 500µl of tween was dispensed into the PBS to form PBS-tween solution.5g of PVP powder was weighed using a weighing balance in aluminum foil and added to the above solution after which equal amount of PVA powder was weighed and added to the mixture; proper agitation and mixing was done using a vortexer. 5mls of BSA was pipetted and added to the solution; finally, 0.2g of Sodium azide was weighed under a biosafety hood in an aluminum foil, added to the solution, properly vortexed and mixed. The final solution labelled with day of preparation and names of those who prepared.

**Preparation of buffer B**

A method by (18) was used in the preparation of buffer B. Briefly, 1000mls of phosphate buffer solution (PBS) was transferred to a sterile glass tube. To this was added, 500ul of tween solution (from thermos scientific), 5g of polyvinyl alcohol (PVA), 5mls of bovine serum albumin solution (BSA), 0.2g sodium azide and 3mls of lyophilized *E.coli* solution. The mixture was incubated overnight at 4^o^C.

IgG positivity rate was determined by subtracting combined sum of background effects and antibody MFI of pooled samples of six Caucasians from UK with no history of exposure to malaria from antibody MFI of blood samples of children born to mothers in Malaria endemic areas.

**Comment #7 : Reviewer 2:** Results
- Figure 1: Review the figure, with good resolution.
- Why does the representation of the graphs differ? Please harmonize
- Specify the number of participants per group (n = ….)
- Association of placental malaria and malaria specific IgG antibodies:
Is there not an effect of both treatment (IPTp) / placental infection with P.f on the level of IgG?
Did all women receive IPTp treatment?
The authors can compare the level of IgG in children from mothers on SP (P.f neg vs P.f pos), and in children from
mothers on PD (P.f neg vs P.f pos).
- Association of malaria specific IgG sub-types and incidence of malaria in the study population
Authors can change “probability of survival” to “probability of fewer occurrence of malaria”

**Response**: Figure was reviewed as guided the corrections are found in our revised submission

- Figure one in the revised manuscript new have better resolutions and more clear graphical representation

-All plots in figure 1 have all been standardized.

-A total of 622 samples from infants were included in this study. Of these, 271 IgG1 results were merged with clinical data base, 305 IgG2 results were merged with clinical data base, 260 IgG3 results were merged with clinical data base and 269 IgG IgG4 results were merged as well.

-All women received IPTq because it is a policy in Uganda for all pregnant. The approved IPTq by the Ugandan Ministry of Health is three doses of SP (common brand is Fansidar) divided across the three trimesters of pregnancy.

-studying effects of both treatment (IPTq) and placental infection with P.f on levels of IgG would be informative but was not the focus of our current study, this can be considered for future studies.

-Yes, all women received IPTq treatment; design of the original study randomized expectant mothers into the two arms of the prophylaxes; DP and SP. DP is the trial drug for IPTq while SP is the approved drug for routine use.

-suggestion of comparing IgG level in children from mothers on SP versus DP according to malaria status is a good one, however this was not done in the current study.

-

**Comment #8; Reviewer 2** Discussion :
The authors can provide explanations for the differences observed only with a few antigens. Do these antigens have any particularities? The results obtained must be well discussed.

**Response:** The study was unique because of the large range of antigen parameter; dropping others means diluting the study. Discussion section has been expounded to capture reviewer’s guidance.

Particularities available include stage of infection where the parasite produced it, purification methods and class in which it belongs.

**Reviewer 3**:

**Comment 1: Reviewer 3:** Major comments
There are numerous grammatical errors throughout the text, figures, and tables. The authors need to have their manuscript reviewed by someone with English as their first language in order to provide a clearer presentation of their findings.

**Response:** This is noted in good faith, keen attention has been put in the revised manuscript to try and minimize grammatical and typing errors. Presentations in terms of tables, graphs will be clearer submitted this time

**Comment 2: Reviewer 3** Minor comments
Abstract and throughout: make sure to specify that you are assaying only for IgG antibodies. For example, the phrase in the Abstract: "Cord concentrations of the erythrocyte binding antigens (EBA140, EBA175 and EBA181) were significantly different." makes it sound like you are detection the actual Pf EBAs, and not the Ig against EBAs.

**Response**: Comment noted; and revised to include reviewer’s guidance; malaria specific

**Comment 3: Reviewer 3**: Throughout: the assay signal for IgG is never translated to a concentration (ug/mL for example), so the authors should not refer to "concentrations" of antibodies in their study. Could replace with a word like "levels"

Response: This is noted, every sentence that has “concentration” has been changed to “level” in the revised manuscript.

**Comment 4: Reviewer 3**: Introduction:
- "Mandatory" for Ugandan women to be enrolled in IPTp?
- Can delete "and consumes most of this essential gas"
- Please modify "most importantly the role of IgG from plasma.." as Ig transfer is one of many protective factors and shouldn't be assigned as the most important
- Can delete "(total and sub-classes)" since this is redundant
- "These anti-malarial immunoglobulins have varied..."
- "Antibodies against merozoite surface proteins are the most expressed in cord blood". You can just say cord blood has high levels of anti-MSP antibodies.
- DP and SP need to be written out at first use.

**Response**: Yes, it a policy that every pregnant mother be given some IPTp at least in every trimester

-“most important” has been deleted as advised by the reviewer.

-“total and sub-classes” deleted as advised

-both reviewer’s input in the last two bullets have been captured in the revised manuscript submitted.

-DP and SP has been quoted right from the beginning of introduction as guided by the reviewer

**Comments 5: Reviewer 3**: Materials and Methods:
- Need to explain specifically how the blood samples were collected, stored short-term (what type of containers, coagulant, fractionated?), and temperature stored at long-term until the IgG assay. It appears plasma was the sample stored long-term.
- Provide company information for all lab chemicals and assay reagents

**Response.**

Primary study where samples for this study were retrieved from was collected during delivery (cord blood) (reference). Collection was done by the midwife, medical officer or obstetrician who conducted delivery or cesarian section. Average of 4mls of accompanying mother’s venous blood was collected as well in an EDTA tube (mother baby pair). Both cord and maternal blood were centrifuged to obtain serum, archived at appropriate temperature, and retrieved for this study. Archival was done in mother-baby pair arrangement, a random-access number generated by the computer was used to track samples in the biobank. Venous blood samples were collected from infants at different time points during first year of life as and when malaria related signs and symptoms such as fever, vomiting or chills appear. Blood samples obtained was prepared and examined for presence of *P.falciparum* parasites; balance archived and used for future study like this one. Obtaining samples at different time points allowed for estimation of antibody decay rates.

**-**All Luminex reagents were made by Luminex Corp, Austin Texas USA.

**Comment 7: Reviewer 3:** Need to provide references for the antigens use in the panel, and more detail on coupling conditions.
- Need to provide software that was used for analysis, and how the Kaplan-Meir curves were created in the software

**Response:**

Graph pad prism was used to draw Kaplan -Meir curves, while box plots were created in STATA (ver 15). To create curve, immunological data (IgG1, IgG2, IgG3 and IgG4) were first merged with clinical data separately. Data was sorted in decreasing levels per IgG sub-type. Data was arranged into 25^th^ percentile (portion containing least values) and 75^th^ percentile (containing largest values) per IgG sub-class. The two groups of infants with varied antibody concentration were compared for occurrence of malaria in the first birth year. Malaria episodes was captured in the clinical data base.

IgG sub-types 1- 4 against 15 *P. falciparum* blood specific antigens and Tetanus Toxoid (TT) was measured in plasma diluted in buffer B. 50µl of bead suspension was added to each well (1,000 beads/region/well) of the 96 plate (Bio-plex pro-flat bottom). The plate was washed, placed on a magnetic block for 2 minutes and the supernatant was then poured off. The beads were washed twice with PBS tween buffer, laid on the magnetic separator for 2 minutes and supernatant poured off.

Reference for the antigens

**Comment 8: reviewer 3:** Results:
- Figure 1: why are all 4 panels not boxplots?
- Figure 1: Missing AMA1 for panel A
- Figure 1: is panel B for IgG2 or IgG3?
- Figure 3: need all x and y axes to be the same among panels and plots. Some x axes appear to be days, and some months. Y axis isn't "Probability of Survival" but "Probability of no malaria infection"
- Figure 3 legend: panel A is IgG1 correct?

**Response.**

All 4 panels in figure one has been revised to box plot

Figure 1 now has AMA1, question of whether panel B is for IgG2 or IgG3 has now been addressed in the revised manuscript.

Yes, panel A in Fig 3 is IgG1.

Mix up of scale in fig 3 has been corrected in the revised manuscript

**Comment 9: reviewer 3**: Discussion:
- "so either of them can be used safely to prevent acquisition of malaria..." the safety of these drugs has nothing to do with IgG antibody levels in women taking them. Please revise.
- "We found no overall association between placental..."
- Please remove the sentence referencing hemozoin as it is incorrect and not relevant here.
- "cofounders such as IgGA"?

**Response:** Statement containing safety of the two drugs has been removed from paragraph 1 of the discussion.

This has been replaced by the statement “We found no overall association between placental”

Statement referring to hemozoin has been deleted as guided by the reviewer
