# Supplementary material for: Effects of anti-malarial prophylaxes on maternal transfer of Immunoglobulin-G (IgG) and association to immunity against Plasmodium falciparum infections among children in a Ugandan birth cohort
Source: PLoS One. 2023 Feb 22;18(2):e0277789. doi: 10.1371/journal.pone.0277789 (PMC9946240; doi:10.1371/journal.pone.0277789)
Supplement: Attachment — Submitted filename: Re-buttal letter (PLOS ONE).docx [file pone.0277789.s009.docx]

Manuscript requirements
Please review your reference list to ensure that it is complete and correct. If you have cited papers that have been retracted, please include the rationale for doing so in the manuscript text, or remove these references and replace them with relevant current references. Any changes to the reference list should be mentioned in the rebuttal letter that accompanies your revised manuscript. If you need to cite a retracted article, indicate the article’s retracted status in the References list and also include a citation and full reference for the retraction notice

Response:

Thank you for the observation; We properly reviewed all citations and noted there was non which was incomplete or retracted from the journal. We noted that only citation number 23 and 25 were repeated and repeats were deleted

Reviewers’ comments

**Reviewer #1, comment#1**

Some of my previous questions/comments were not addressed in this revision which I will restate below. Due to the many factors that can influence malaria risk I still think a multivariate Cox regression is useful to understand the association.

**Response:** As guided by the reviewer, A multivariate cox regression using Breslow method for ties of the survival data was used to do this analysis. Failure variable was fever in a child in the first birth year, failure values were 3,4,5 which were episodes of fever while time to event was measured in days. The covariates included in the analysis are mother’s prophylactic arm (SP or DP), Insecticide treated mosquito use (used or not), placental malaria by histology (negative or positive) and total IgG levels against 16 malaria specific antigens

**Reviewer #1, comment#2**

A total of 16 Ig-G subtypes were used in the comparison and p-values need to be adjusted for multiple testing. I am not familiar with this research area, but would it be meaningful to combine all Ig-G subtypes into one variable in the analysis? This way multiple testing is not a concern, and the main message will be easier to see. Right now, there are too many comparisons which makes the result hard to interpret.

**Response**: Thank you for your keen observation; to add meaning to p-value during multivariate cox regression analysis, hazard ratio and z values were added to bring more meaning into the results.

**Reviewer #1, comment#3**

The conclusion from the survival model is that “antibody titer against P.falciparum may be more of a marker of exposure to previous malaria infections among infants from malaria endemic areas as opposed to their protective roles” which is interesting and that is why I recommended using placental malaria status as the grouping variable. If your conclusion is true, I would expect to see higher malaria rates among those positive for placental malaria. The Cox model may also help to tease apart the effects of placental malaria and Ig-G levels.

**Response:** we take note of this recommendation; In the cox regression analysis, Prescence of placental malaria by histology during delivery did not increase risk of developing fever in a child’s first birth year as there was no difference in number of fever episodes in children born to mothers with either placental malaria status (HR1. 1.3394,p=0.264 (table 3). We went further examined if difference in income levels affects birth weight of a child, time to first malaria incidence and number of malaria episodes. All three outcomes were aligned to mother’s economic status in a grouped column of graph pad prism. Two-way Analysis of Variance was used to compare difference in the mean values of the three groups across the three outcomes

**Reviewer #3**: The authors provide an improved version of the manuscript which is much easier to read and comprehend. A few minor issues need to be addressed:

**Reviewer #3,comment #1**

In Methods, the authors need to add company information for all their chemicals, reagents, and other labware

**Response**: We take note of your guidance; company names for almost reagents and chemicals used has been included in the revised manuscript

**Reviewer #3,comment #2**

Concentrations of reagents such as secondary antibody and PE-conjugate used for the assays need to be included in Methods

**Response:** This has been factored in the revised manuscript in this statement “50µl of a secondary antibody specific for an IgG sub-type **(IgG1, IgG2, IgG2, IgG3 and IgG4) with concentration of 1in1000 for IgG1,IgG2 and IgG4 while secondary antibody against IgG4 was diluted at 1in 2000** in dilution buffer, Using a multichannel pipette, **50µl of 1in 200 R-Phycoerythrin**-conjugate AffinPure F (ab’) goat anti-human IgG

**Reviewer #3,comment #2**

For Figures 1 and 2, include labels for the y axis on each panel. Perhaps on the x axis, can state "Placental Malaria", "No Placental Malaria", rather than "No", "Yes"

**Response**: y axes have been labelled for all graphs as “log transformed IgG levels”; X-axis labelling has been adjusted in the revised manuscript.
