# Supplementary material for: Effects of anti-malarial prophylaxes on maternal transfer of Immunoglobulin-G (IgG) and association to immunity against Plasmodium falciparum infections among children in a Ugandan birth cohort
Source: PLoS One. 2023 Feb 22;18(2):e0277789. doi: 10.1371/journal.pone.0277789 (PMC9946240; doi:10.1371/journal.pone.0277789)
Supplement: Attachment — Submitted filename: Rebuttal_Response to the Authors comments (true).docx [file pone.0277789.s010.docx]

Review Comments to the Author

Please use the space provided to explain your answers to the questions above. You may also include additional comments for the author, including concerns about dual publication, research ethics, or publication ethics. (Please upload your review as an attachment if it exceeds 20,000 characters)

Reviewer #1: Cox model: why were only subjects with >=3 episodes of fevers considered as a failure instead of occurrence of malaria as in Figure 4? How were subjects with 1 and 2 episodes handled in the model?

**Response: Thank you for the observation; we have dropped the number of fever episodes as a failure event and replaced it with malaria occurrence in a child throughout the observation period, failure event is now occurrence of malaria. This is adjusted for in the copy with tracked changes and the revised version. Failure values are 1,2,3,4,5**

Family socioeconomic status and placental malaria status need to be adjusted for in the cox model. There are too many IgG variables in the model. Are different IgG levels correlated? If not, maybe better to fit each IgG in the model in turn and adjust the p-values for multiple testing.

**Response: Your comment is noted and addressed; all covariates (parasite prevalence by microscopy during pregnancy, malaria episodes during pregnancy, choice of IPT, placental malaria and total IgG response against malaria specific antigens) had their P-values adjusted for using Benjamin-Hoceberg correction for multiple testing in excel (Table 3)**

Table 3 needs to show variable description instead of variables names used in the coding. What is the overall P value? Is it likelihood ratio test of all the variables in the model? The likelihood ratio test should test for all the IgG related variables only.

**Response: Full names of variables have been captured below the table under the key. Variables full names are too long to fit in the variable column; readers will be referred to the key below table 3. The first 5 columns of table 3; including overall P value has been deleted as advised by reviewer 3; this adresses your concerns as well.**

It is not clear what is Figure 3A showing. Do the three tick marks on the x-axis in each subplot denote different outcome? What is the y-axis? Birth weight cannot be close to 0. Also, it is not clear how the 2-way ANOVA was set up? If birth weight of a child, time to first malaria incidence and number of malaria episodes are the outcomes and mother’s economic status is a factor what is the other factor?

**Response: Comments well noted; time to 1^st^ malaria incidence and number of malaria incidence have been dropped off the variable list for the analysis; only birth weight has been maintained. Two way ANOVA in a grouped column graph was also dropped off as the analysis method; only mean data plot for birth weight versus economic status was maintained in the analysis (fig 3).**

Result section on Kaplan-Meir survival curve, why was it called “reciprocal hazard ratio”? Higher level was associated with higher risk. Shouldn’t the value presented just be the hazard ratio for higher levels of IgG?

**Thank you for the keen observation and guidance; It was indeed in error to refer to it as reciprocal Hazard ratio; it has been corrected to read Hazard ratio as it is the right statistical language.**

Reviewer #2: (No Response)

Reviewer #3: The authors still need to arrange some elements for easier interpretation. Table 3 should have defined rows and columns (like Tables 1 and 2) and the first 5 rows ("Time variable...." to "Overall P...") need to be deleted. In Figure 3, the two plots need clear labeling on the x and y-axes. For A, I assume the y-axis is birthweight, but what are the three categories on the x-axis? For B, I assume the x-axis is time to first malaria episode?

**Table 3 titled “multivariate cox regression of malaria occurrence in the first birth year of children associated with selected maternal characteristics/exposure variables” has clearly and well-defined titles within each column. For titles of rows, short forms, and acronyms of the full names have been used. Full names are captured under the key below the table. First five rows of table 3 deleted as guided. Figure 3A was removed as guided by the 1^st^ reviewer, For Figure 3B, the two variables of time to first malaria and number of malaria episodes were dropped in the data mean set analysis; only birth weight was sustained as variable of comparison with income status. Figure 3 is now clearer and straight forward**
