# Supplementary material for: Effects of anti-malarial prophylaxes on maternal transfer of Immunoglobulin-G (IgG) and association to immunity against Plasmodium falciparum infections among children in a Ugandan birth cohort
Source: PLoS One. 2023 Feb 22;18(2):e0277789. doi: 10.1371/journal.pone.0277789 (PMC9946240; doi:10.1371/journal.pone.0277789)
Supplement: Attachment — Submitted filename: 4TH response_PLOS ONE.docx [file pone.0277789.s011.docx]

Reviewer #1, Qn 1: “Placental malaria prevalence”- is this a binary (presence/absence) variable or a continuous variable?

Yes, placental malaria is a binary variable and was measured as a Yes or No. This has been clearly indicated in the revised Manuscript.

**Reviewer # 1, Qn 2:** Cox regression: “total IgG”- is the sum of IgG across IgG1, IgG2, IgG3 and IgG4? There are many IgG variables in the model and there may be collinearity. My suggestion is to include only IgG specific to one antigen at a time as a predictor in the cox regression while adjusting for other non-IgG covariates. The p-values of the IgG variables from all the different cox models should be adjusted for multiple testing.

Response: Yes, we agree to the reviewer’s suggestion, and we included only IgG specific to one antigen at a time in building the Cox regression model while adjusting for non-IgG covariates at each time. The Cox regression model was built using back ward elimination method. For each IgG, the levels were grouped into percentiles, that is 25^th^, 50^th^ and 75^th^ . The effects of different levels of the IgG (25^th^,50^th^ and 75^th^ percentiles) on the incidence of malaria among study participants were assessed. The levels of the IgG at the 75^th^ percentile was found to consistently have higher Hazard ratios and significant confidence intervals. Therefore, the levels of the IgG at the 75^th^ percentile were used in building the Cox regression model. The P-values of the IgG variables in the Cox regression models were adjusted for Multiple testing. The non-IgG covariates included birth weight (<2500 grams, >2500grams), maternal malaria incidence (No malaria detected, and malaria detected), Maternal wealth index (poorest, middle, and least poor), gravidity category (1-3 and 4+). Cofounding was assessed in the Cox regression model and any variable that had the percentage difference of more than 10% between the crude and adjusted Hazard ratios was considered a cofounder. In the model building, the covariates that had p value of ≤ 0.2 in the bivariate analysis were included in the model.

**Reviewer #1,Qn 3**:Why wasn’t mother’s economic status included as a covariate in the model?

**Response**: Yes, we agree with the reviewer’s suggestion, in the revised manuscript, we have included Mother’s wealth index (Poorest, middle and least poor)

**Reviewer #1, Qn4,** Cox regressions adjusting for potential confounders are more informative than Kaplan-Meir survival analysis. The section “Probability of survival to “probability of fewer occurrence of malaria”” seems to be superfluous.

Response: Yes, we agree with the reviewer’s suggestion and in the revised manuscript, we developed a Cox regression model and adjusted for multiple testing in the IgG covariates and for cofounding in all other variables including the mother’s characteristics. .

**Reviewer #1, Qn5**: The section “Effects of Economic status on birth outcomes and risk of malaria incidence”- the only association shown is with birth weight, which is not relevant to the main topic of this paper. If there is no association with malaria incidence or IgG levels, the entire section can go. Please go through the paper carefully and remove any language related to this analysis.

**Response: Guidance well noted; section on “Effects of Economic status on birth outcomes” has been removed as advised in the revised manuscript. We included Mother’s wealth category in the Cox regression model and assessed if it cofounds the estimation of malaria incidence amongst study participants**

Reviewer #2: The authors have done an excellent job. I read with great interest the responses to the various comments from reviewers.
A minor error found in the “laboratory procedures”: 50μl of a secondary antibody specific for an IgG subtype (IgG1, IgG2, IgG2, IgG3 and IgG4)...IgG2 is repeated twice.

Response: Thank you so much for your keen observation; IgG2 was indeed repeated. This has been corrected in the revised manuscript.

Reviewer #3: Authors should attempt to further clean up their figures and will need to submit publication-quality for the final version.

Response: Yes, we agree with the reviewer’s comments, and we have improved the quality of the figures in the revised manuscript.
